# Assessment of autozygosity in Nellore cows (*Bos indicus*) through high-density SNP genotypes

**DOI:** 10.3389/fgene.2015.00005

**Published:** 2015-01-29

**Authors:** Ludmilla B. Zavarez, Yuri T. Utsunomiya, Adriana S. Carmo, Haroldo H. R. Neves, Roberto Carvalheiro, Maja Ferenčaković, Ana M. Pérez O'Brien, Ino Curik, John B. Cole, Curtis P. Van Tassell, Marcos V. G. B. da Silva, Tad S. Sonstegard, Johann Sölkner, José F. Garcia

**Affiliations:** ^1^Departamento de Medicina Veterinária Preventiva e Reprodução Animal, Faculdade de Ciências Agrárias e Veterinárias, UNESP – Univ Estadual PaulistaJaboticabal, São Paulo, Brazil; ^2^GenSys Consultores AssociadosPorto Alegre, Rio Grande do Sul, Brazil; ^3^Departamento de Zootecnia, Faculdade de Ciências Agrárias e Veterinárias, UNESP – Univ Estadual PaulistaJaboticabal, São Paulo, Brazil; ^4^Department of Animal Science, Faculty of Agriculture, University of ZagrebZagreb, Croatia; ^5^Division of Livestock Sciences, Department of Sustainable Agricultural Systems, BOKU - University of Natural Resources and Life SciencesVienna, Austria; ^6^Animal Genomics and Improvement Laboratory, United States Department of Agriculture, Agricultural Research ServiceBeltsville, MD, USA; ^7^Bioinformatics and Animal Genomics Laboratory, Embrapa Dairy CattleJuiz de Fora, Minas Gerais, Brazil; ^8^Laboratório de Bioquímica e Biologia Molecular Animal, Departamento de Apoio, Produção e Saúde Animal, Faculdade de Medicina Veterinária de Araçatuba, UNESP – Univ Estadual PaulistaAraçatuba, São Paulo, Brazil

**Keywords:** *Bos indicus*, runs of homozygosity, selection, cattle, fertility, disease resistance

## Abstract

The use of relatively low numbers of sires in cattle breeding programs, particularly on those for carcass and weight traits in Nellore beef cattle (*Bos indicus*) in Brazil, has always raised concerns about inbreeding, which affects conservation of genetic resources and sustainability of this breed. Here, we investigated the distribution of autozygosity levels based on runs of homozygosity (ROH) in a sample of 1,278 Nellore cows, genotyped for over 777,000 SNPs. We found ROH segments larger than 10 Mb in over 70% of the samples, representing signatures most likely related to the recent massive use of few sires. However, the average genome coverage by ROH (>1 Mb) was lower than previously reported for other cattle breeds (4.58%). In spite of 99.98% of the SNPs being included within a ROH in at least one individual, only 19.37% of the markers were encompassed by common ROH, suggesting that the ongoing selection for weight, carcass and reproductive traits in this population is too recent to have produced selection signatures in the form of ROH. Three short-range highly prevalent ROH autosomal hotspots (occurring in over 50% of the samples) were observed, indicating candidate regions most likely under selection since before the foundation of Brazilian Nellore cattle. The putative signatures of selection on chromosomes 4, 7, and 12 may be involved in resistance to infectious diseases and fertility, and should be subject of future investigation.

## Introduction

Autozygosity is the homozygote state of identical-by-descent alleles, which can result from several different phenomena such as genetic drift, population bottleneck, mating of close relatives, and natural and artificial selection (Falconer and Mackay, 1996; Keller et al., 2011; Curik et al., 2014). In the past 20 years, the heavy use of relatively low number of sires in Brazilian Nellore breeding programs (*Bos indicus*) is deemed to have mimicked all these triggers of autozygosity, especially considering the increasing use of artificial insemination over the decades. As inbreeding has been incriminated in reduced fitness and reproductive performance in other cattle populations under artificial selection (Bjelland et al., 2013; Leroy, 2014), avoidance of mating of close relatives is a typical practice of many Nellore breeders. Therefore, there is a growing interest in characterizing and monitoring autozygosity in this breed to preserve genetic diversity and allow the long-term sustainability of breeding programs in Brazil.

Evidence from whole-genome sequencing studies in humans indicate that highly deleterious variants are common across healthy individuals (MacArthur et al., 2012; Xue et al., 2012), and although no such systematical survey has been conducted in cattle to the present date, it is highly expected that unfavorable alleles also segregate in cattle populations. Therefore, the use of ever-smaller numbers of animals as founders is expected to inadvertently increase autozygosity of such unfavorable alleles (Szpiech et al., 2013), potentially causing economic losses.

Recently, the use of high-density single nucleotide polymorphism (SNP) genotypes to scan individual genomes for contiguous homozygous chromosomal fragments has been proposed as a proxy for the identification of identical-by-descent haplotypes (Gibson et al., 2006; Lencz et al., 2007). As the length of autozygous chromosomal segments is proportional to the number of generations since the common ancestor (Howrigan et al., 2011), the identification of runs of homozygosity (ROH) can reveal recent and remote events of inbreeding, providing invaluable information about the genetic relationships and demographic history of domesticated cattle (Purfield et al., 2012; Ferenčaković et al., 2013a; Kim et al., 2013). Also, given the stochastic nature of recombination, the occurrence of ROH is highly heterogeneous across the genome, and hotspots of ROH across a large number of samples (hereafter referred as common ROH) may be indicative of selective pressure. Moreover, the fraction of an individual's genome covered by ROH can be used as an estimate of its genomic autozygosity or inbreeding coefficient (McQuillan et al., 2008; Curik et al., 2014).

Here, we investigated the occurrence of ROH in high-density SNP genotypes in order to characterize autozygosity in the genomes of a sample of 1,278 Nellore cows under artificial selection for weight, carcass and reproductive traits. We aimed at characterizing the distribution of ROH length and genome-wide levels of autozygosity, as well as detecting common ROH that may be implicated in past events of selection.

## Materials and methods

### Ethical statement

The present study was exempt of the local ethical committee evaluation as genomic DNA was extracted from stored hair samples of animals from commercial herds.

### Genotyping and data filtering

A total of 1,278 cows were genotyped with the Illumina® BovineHD Genotyping BeadChip assay (HD), according to the manufacturer's protocol (http://support.illumina.com/array/array_kits/bovinehd_dna_analysis_kit.html). These animals comprised part of the genomic selection reference population from a commercial breeding program. These dams were born between 1993 and 2008, being under routine genetic evaluation for weight, carcass and reproductive traits by the DeltaGen program, an alliance of Nellore cattle breeders from Brazil. Data filtering was performed using *PLINK v1.07* (Purcell et al., 2007), and markers were removed from the dataset if GenTrain score lower than 70% or a call rate lower than 98% was observed. All genotyped samples exhibited call rates greater than 90%, thus no animals were filtered from further analyses. Minor allele frequency (MAF) was not used as an exclusion criterion in this analysis, so that the detection of homozygous segments was not compromised. Both autosomal and X-linked markers were included.

### Estimates of genomic individual autozygosity

Genomic autozygosity was measured based on the percentage of an individual's genome that is covered by ROH. Stretches of consecutive homozygous genotypes were identified for each animal using *SNP & Variation Suite v7.6.8* (Golden Helix, Bozeman, MT, USA http://www.goldenhelix.com), and chromosomal segments were declared ROH under the following criteria: 30 or more consecutive homozygous SNPs, a density of at least 1 SNP every 100 kb, gaps of no more than 500 kb between SNPs, and no more than 5 missing genotypes across all individuals. In order to account for genotyping error and avoid underestimation of long ROH (Ferenčaković et al., 2013b), heterozygous genotype calls were allowed under conditions where there were 2 heterozygous genotypes for ROH ≥ 4 Mb, or no heterozygous genotypes for ROH < 4 Mb. Autozygosity was estimated according to McQuillan et al. (2008):

$$F_{ROH}=\frac{\sum_{j\;=\;1}^{n}L_{ROH_{j}}}{L_{total}}$$

Where *L*_*ROH*__*j*_ is the length of ROH *j*, and *L*_*total*_ is the total size of the genome covered by markers, calculated from the sum of inter-marker distances in the UMD v3.1 assembly. In order to facilitate comparisons with other studies, *F*_*ROH*_ was calculated using both the genome size based on autosomal and autosomal + X chromosomes. For each animal, *F*_*ROH*_ was calculated based on ROH of different minimum lengths: 0.5, 1, 2, 4, 8 or 16 Mb, representing autozygosity events that occurred approximately 100, 50, 25, 13, 6, and 3 generations in the past, respectively (Howrigan et al., 2011; Ferenčaković et al., 2013b). Additionally, chromosome-wise *F*_*ROH*_ was also computed.

An alternative measure of autozygosity was obtained by computing the diagonal elements of a modified realized genomic relationship matrix (VanRaden, 2008; VanRaden et al., 2011), calculated as:

$$G=\frac{ZZ^{\prime }}{2\sum_{l\;=\;1}^{n}p_{l}(1-p_{l})}$$

Where *Z* is a centered genotype matrix and *p*_*l*_ is the reference allele frequency at locus *l*. Matrix *Z* is obtained by subtracting from the genotype matrix *M* (with genotype scores coded as 0, 1 or 2 for alternative allele homozygote, heterozygote, and reference allele homozygote, respectively) the matrix *P*, whose elements of column *l* are equal to 2*p*_*l*_. The diagonal elements of *G* (*G*_*i*, *i*_) represent the relationship of an animal with itself, and thus encapsulate autozygosity information. Following VanRaden et al. (2011), *G*_*i*, *i*_ can provide a more suitable proxy for the pedigree-based inbreeding coefficient when assuming *p*_*l*_ = 0.5, rather than using base population allele frequencies estimates (which could be difficult to estimate especially in absence of complete pedigree data). Thus, matrix *G* was computed using allele frequencies fixed at 0.5.

### Detection of common runs of homozygosity

Chromosomal segments presenting ROH hotspots were defined as ROH islands or common ROH. In order to identify such genomic regions, we used two different strategies. First, we used the clustering algorithm implemented in *SNP & Variation Suite v7.6.8*, which identifies clusters of contiguous set of SNPs with size > *s*_*min*_, where every SNP has at least *n*_*min*_ samples presenting a run. Clusters were identified based on a fixed minimum cluster size of *s*_*min*_ = 0.5 Mb for varying minimum number of samples: 127 (10%), 255 (20%), 319 (25%), and 639 (50%). In order to assess the sensitivity of the algorithm to parameter settings in ROH detection, we repeated the analysis using minimum numbers of 30 or 150 SNPs in a run, maximum gap sizes of 100 kb or 500 kb, and 0 or 2 heterozygous genotypes as variable parameters.

Alternatively, we calculated locus autozygosity (*F*_*L*_) following Kim et al. (2013). Briefly, for each SNP, animals were scored as autozygous (1) or non-autozygous (0) based on the presence of a ROH encompassing the SNP. Then, the locus autozygosity was simply computed as:

$$F_{L}=\frac{\sum_{i\;=\;1}^{n}S_{i}}{n}$$

where *S*_*i*_ is the autozygosity score of individual *i*, and *n* is the number of individuals. In essence, *F*_*L*_ represents the proportion of animals with scores equal to 1 (i.e., that present a ROH enclosing the marker), thus it summarizes the level of local autozygosity in the sample.

## Results and discussion

### Distribution of ROH length

After filtering, 668,589 SNP marker genotypes across 1,278 animals were retained for analyses. The average, median, minimum and maximum ROH length detected across all chromosomes were 1.26, 0.70, 0.50, and 70.91 Mb, respectively, suggesting this specific Nellore cattle population experienced both recent and remote autozygosity events. Segments as large as 10 Mb are traceable to inbreeding that occurred within the last five generations (Howrigan et al., 2011), and a total of 942 samples (73.7%) presented at least one homozygous fragment larger than 10 Mb. Therefore, it is likely that these long ROH are signatures of the extended use of recent popular sires.

### Distribution of genome-wide autozygosity

**Figure 1:**
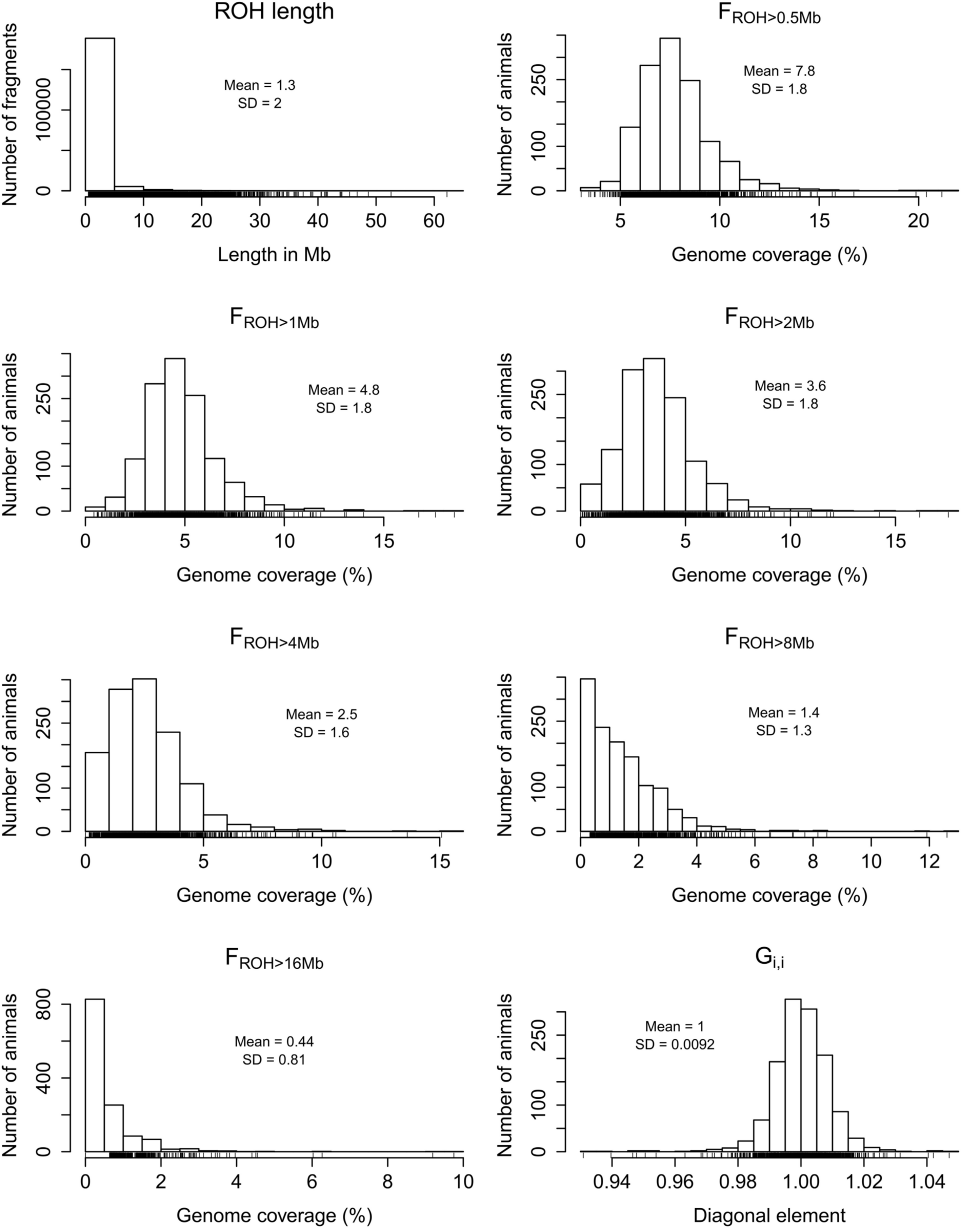
**Frequency distributions of all detected runs of homozygosity (ROH) across samples, percentage of autosomal genome coverage by ROH (*F*_*ROH*_) of different minimum lengths (>0.5, >1, >2, >4, >8, and >16 Mb), and diagonal elements of the realized genomic relationship matrix (*G*_*i*, *i*_)**.

The distributions of *G*_*i*, *i*_ and *F*_*ROH*_ based on autosomal ROH of different minimum lengths (>0.5, >1, >2, >4, >8 or >16 Mb) are shown in Figure 1. Although the inclusion of the X chromosome did not cause substantial differences in the calculation of genome-wide *F*_*ROH*_ (Supplementary Figure S1), we focused on the estimates using only autosomes for the ease of comparison with other studies. The skewness of the autosomal *F*_*ROH*_ distribution increased as the minimum fragment length increased, ranging from 1.56 for *F*_*ROH* > 0.5 *Mb*_ to 3.98 for *F*_*ROH* > 16 *Mb*_. The number of animals with *F*_*ROH*_ = 0 also increased as the minimum ROH length increased, starting at 12 (0.94%) for *F*_*ROH* > 2 *Mb*_ and increasing to 827 (64.71%) for *F*_*ROH* > 16 *Mb*_. Under the assumption of the relationship between ROH length and age of autozygosity, these findings show that varying the minimum ROH length in the calculation of *F*_*ROH*_ can be useful to discriminate animals with recent and remote autozygosity.

**Figure 2:**
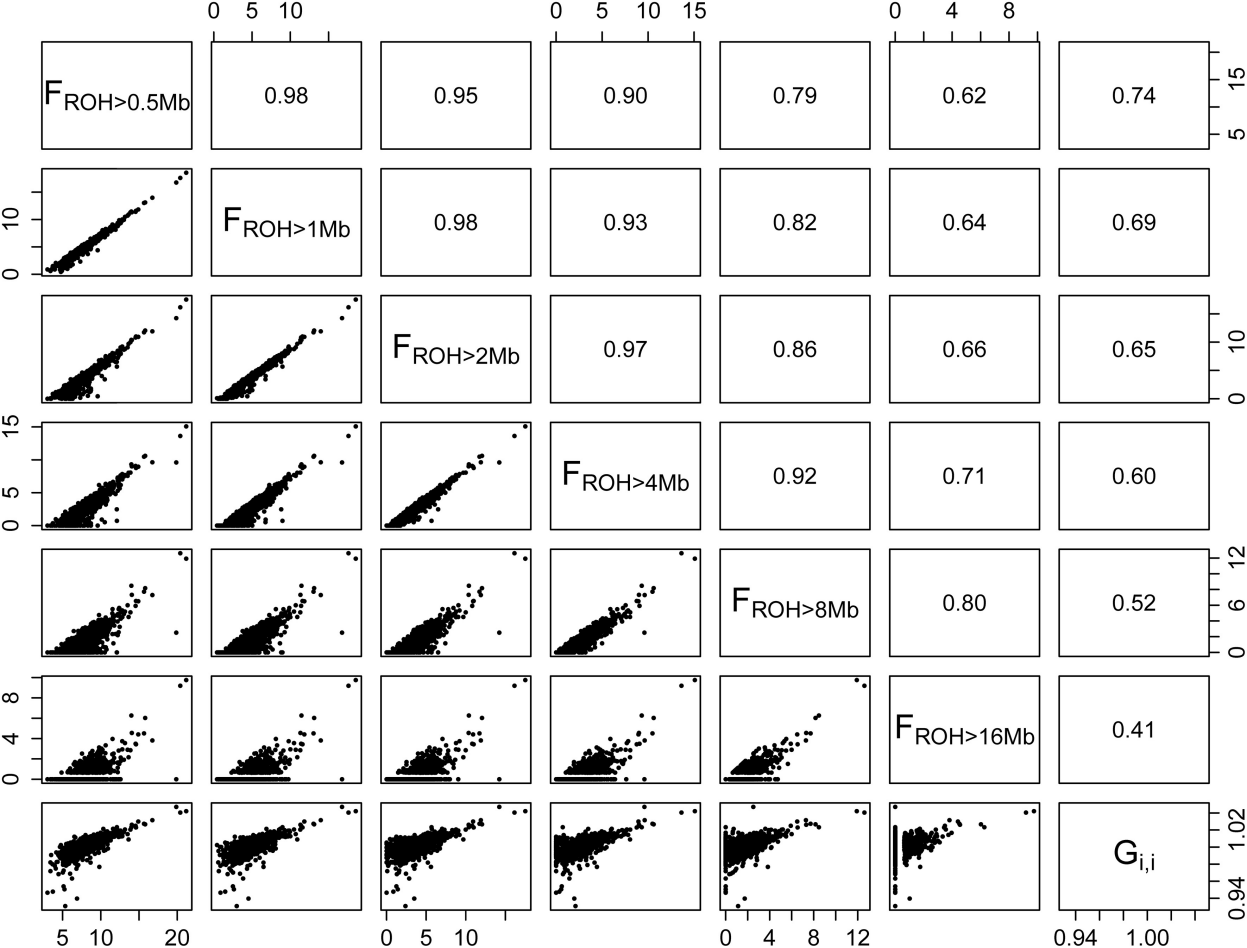
**Scatterplots (lower panel) and correlations (upper panel) of percentage of autosomal genome coverage by runs of homozygosity (*F*_*ROH*_) of different minimum lengths (>0.5, >1, >2, >4, >8, and >16 Mb) and diagonal elements of the realized genomic relationship matrix (*G*_*i*, *i*_)**. The last column of panels on the right indicates that the correlation between *F*_*ROH*_ and *G*_*i*, *i*_ decreases as a function of minimum fragment size.

As shown in Figure 2, the correlation between autosomal *F*_*ROH* > 1 *Mb*_ and *G*_*i*, *i*_ (*r* = 0.69) was close to the ones reported by Ferenčaković et al. (2013b) for the comparison between *F*_*ROH* > 1 *Mb*_ derived from the HD panel and pedigree estimates in Brown Swiss (*r* = 0.61), Pinzgauer (*r* = 0.62), and Tyrol Gray (*r* = 0.75). Similar correlations were observed when the X chromosome was included in the analysis (Supplementary Figure S2). McQuillan et al. (2008) also reported correlations between *F*_*ROH*_ and pedigree estimates in human European populations ranging from 0.74 to 0.82. Considering that VanRaden (2008) proposed *G* as a proxy for a numerator relationship matrix obtained from highly reliable and recursive pedigree data, we expect that the correlations found for *G*_*i*, *i*_ are fair approximations to the ones we would have found if complete pedigree data was available.

In the present study, correlations between *F*_*ROH*_ and *G*_*i*, *i*_ decreased as a function of different ROH length (Figure 2). This may be due to the properties of the *G* matrix, which is based on individual loci, whereas *F*_*ROH*_ is based on chromosomal segments. Ferenčaković et al. (2013b) showed that medium density SNP panels, such as the Illumina® BovineSNP50, systematically overestimate *F*_*ROH*_ when segments shorter than 4 Mb are included in the calculations, while the Illumina® BovineHD panel is robust for the detection of shorter segments. Hence, although the inclusion of short length ROH in the calculation of *F*_*ROH*_ may be desirable for autozygosity estimates accounting for remote inbreeding, there is a compromise between SNP density, minimum ROH length and false discovery of ROH. Since the HD panel allows for the detection of short ROH, in this section we focused on the results obtained with *F*_*ROH* > 1 *Mb*_ as it presented the second highest correlation with *G*_*i*, *i*_ and is comparable with previous studies.

The minimum, average, median, and maximum autosomal *F*_*ROH* > 1 *Mb*_ across all animals were 0.43, 4.79, 4.58, and 18.55%, respectively. The animal presenting the highest autozygosity value (18.55%) exhibited 69 ROH > 1 Mb encompassing 465.66 Mb of the total autosomal genome extension covered by markers (2.51 Gb), with a mean ROH length of 6.75 ± 9.20 Mb, and a maximum segment length of 43.79 Mb. The least inbred animal presented 8 ROH > 1 Mb, summing up only 10.72 Mb, with an average length of 1.34 ± 0.46 Mb and a maximum of 2.43 Mb.

The coefficient of variation (here denoted as the ratio of the standard deviation to the mean) of the *F*_*ROH* > 1 *Mb*_ distribution was 37.5%, indicating moderate variability in autozygosity levels in this sample. In spite of the average genome coverage by ROH of 4.58% may seem to indicate moderate inbreeding levels for classical standards, it has to be considered that incomplete pedigree data usually fails to capture remote inbreeding, so that traditional inbreeding estimates based on pedigree are only comparable with *F*_*ROH*_ calculated over large ROH lengths, which in the present study were close to 0%.

Compared to other cattle populations, this sample of Nellore cows presented a lower average autozygosity. For instance, Ferenčaković et al. (2013b) reported average autosomal *F*_*ROH* > 1 *Mb*_ of 15.1%, 6.2%, and 6.6% for samples of the *Bos taurus* breeds Brown Swiss, Pinzgauer, and Tyrol Gray, respectively. Also, the effective population size estimated for this Nellore sample was approximately 362 animals (Supplementary Material), which is consistent with the low genome average LD reported by other studies (McKay et al., 2007; Espigolan et al., 2013; Pérez O'Brien et al., 2014) and indicative of a non-inbred population.

### Distribution of chromosome-wise autozygosity

**Figure 3:**
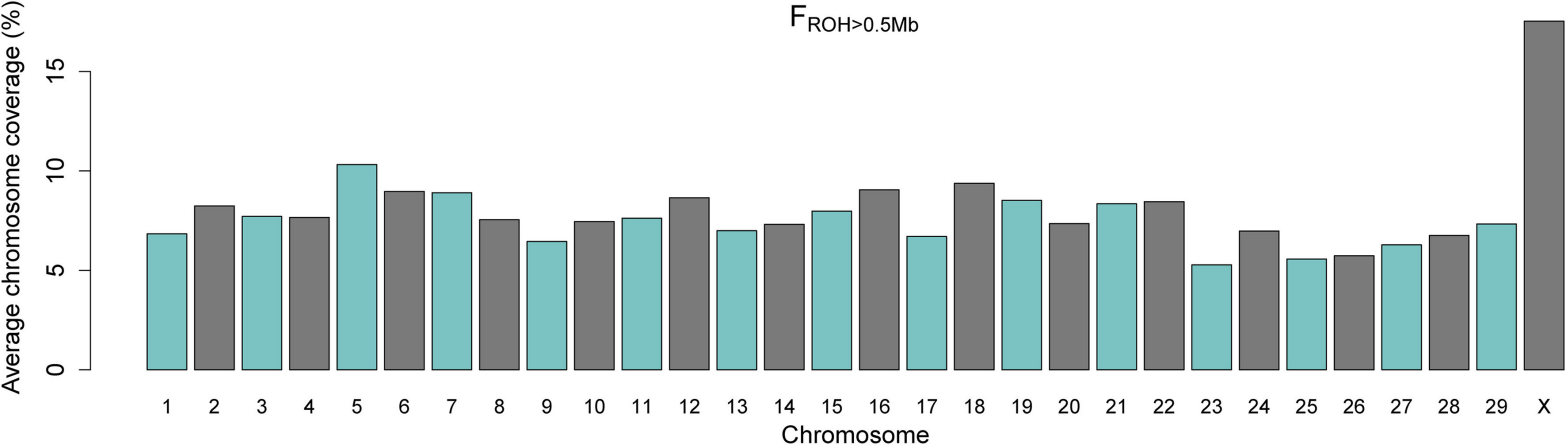
**Barplot of average percentage of chromosome coverage by runs of homozygosity (*F*_*ROH*_) of minimum length of 0.5 Mb**.

The averages of the chromosome-wise *F*_*ROH* > 0.5 *Mb*_ across samples are shown in Figure 3. Chromosome X exhibited a substantially higher average autozygosity when compared to the autosomes. Importantly, we found no evidence for a smaller effective population size for the X chromosome in comparison to the autosomal genome (Supplementary Material). This may be due to the mode of inheritance of the X chromosome, which is hemizygous in the male lineage and therefore more susceptible to bottlenecks and drift even under assumptions of balanced numbers of males and females (Gottipati et al., 2011).

An alternative explanation is that the gene content and the sex-specific copy number of the X chromosome is under stronger selective pressure in comparison to autosomal DNA (Hammer et al., 2010; Deng et al., 2014). In both hypotheses, this higher autozygosity may reflect historical and demographical events. In the early 20th century, when more frequent importation of Nellore cattle to Brazil was initiated, the indigenous herds mainly consisted of descendants from taurine (*Bos taurus*) cattle imported since the late 15th century after the discovery of America (Ajmone-Marsan et al., 2010). In spite of the use of taurine dams for breeding during the early establishment of Nellore cattle in Brazil, the decades that followed were marked by intense backcrossing to Nellore bulls, causing most of the taurine contribution to be swept out from the Nellore autosomal genome (Utsunomiya et al., 2014). However, it is well-established that taurine mitochondrial DNA is prevalent in Nellore cattle, as it is a strict maternal contribution (Meirelles et al., 1999). Therefore, the X chromosome may have experienced a greater drift than the autosomal genome due to limited number of founders. The levels of taurine introgression still segregating in the X chromosome in this herd remain unclear.

### Identification of common ROH

Table 1 presents the results obtained from the ROH clustering analysis. The algorithm was robust in respect to gap size between SNPs, but substantial differences were observed when the number of consecutive SNPs and the number of heterozygous genotypes were modified. Few common ROH were identified even when the minimum number of samples in the cluster was 10%, indicating that ROH distribution is not uniform across the genome. In fact, despite of the occurrence of 99.98% of the SNPs within a ROH of at least one individual, only 19.37% markers were encompassed by ROH observed in 10% or more of the samples. This finding is similar to that reported by Ferenčaković et al. (2013b), and is consistent with the stochastic nature of meiotic recombination. This suggests that the ongoing selection for weight, carcass and reproductive traits in this population has not yet created detectable ROH-based selection signatures related to production.

**Table 1:** **Detection of common runs of homozygosity according to different number of consecutive SNPs, percentage of animals, gap size, and number of heterozygous genotypes**.

**Gap size**	**30 SNPs**				**150 SNPs**				**Heterozygotes**
	**10%**	**20%**	**25%**	**50%**	**10%**	**20%**	**25%**	**50%**	
100 Kb	437	106	57	9	186	29	12	1	0
	471	288	183	29	365	91	47	7	2
500 Kb	479	126	76	13	193	32	14	1	0
	768	334	214	45	375	96	50	7	2

**Figure 4:**
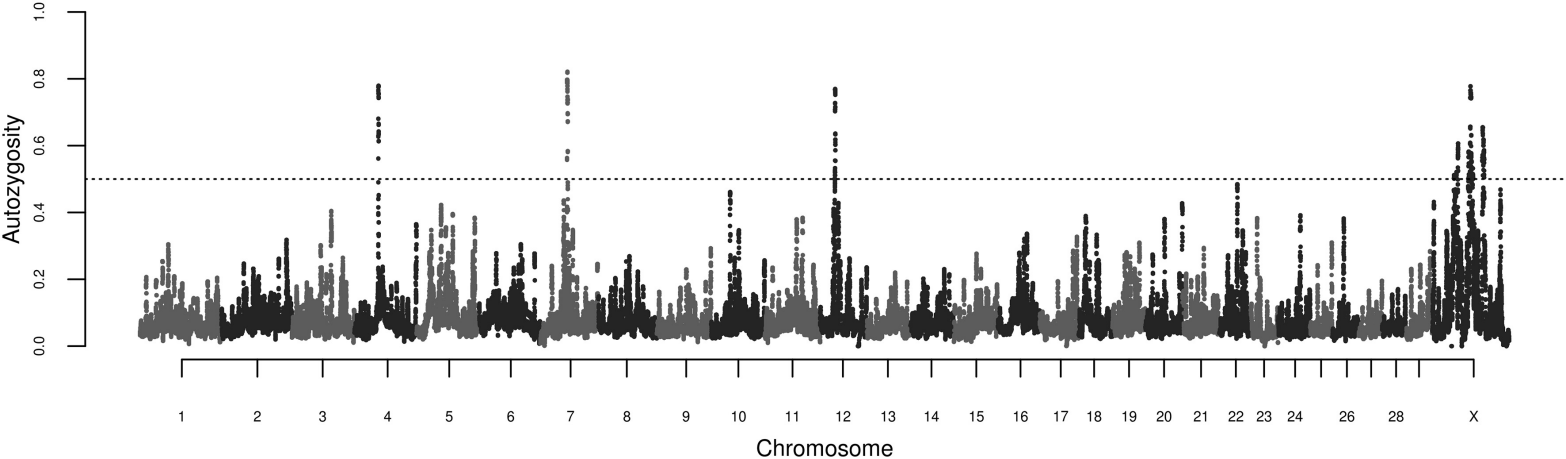
**Manhattan plot of genome-wide locus autozygosity in Nellore cows**. The dashed line represents the 50% threshold.

The calculations of locus autozygosity were consistent with the cluster analysis using 150 SNPs and 2 heterozygous genotypes, regardless of permitted gap size (Figure 4). Seven distinct genomic regions, four of them on chromosome X, presented strong hotspots of autozygosity, where over half of the samples (*n* = 639) contained a ROH. The common ROH on the X chromosome are difficult to be discussed as they span several millions of bases, encompassing hundreds of genes and making functional explorations unfeasible. Besides, the assembly status of X chromosome is poorer than the autosomal ones. Hence, we focused on the three autosomal regions on chromosomes 4, 7, and 12. The three regions were relatively short, ranging from 0.73 to 1.43 Mb. For this range of ROH length, the expected number of generations since the common ancestor is estimated between 35 and 69 (Howrigan et al., 2011). Assuming a cattle generation interval of 5 years, these inbreeding events may have occurred between 175 and 345 years ago. Although this estimate does not account for birth date and overlapping generations, these remote autozygosity events are likely to predate the foundation of the Nellore breeding programs, and therefore expected to be related to natural selection, random drift or population bottlenecks.

The most autozygous locus was found at chromosome 7:51605639-53035752. This region was previously reported in genome-wide scans for signatures of selection in cattle through the comparison of *Bos taurus* and *indicus* breeds via F_*ST*_ analysis (Bovine HapMap Consortium, 2009; Porto-Neto et al., 2013) and was detected as a ROH hotspot in an analysis of three taurine and indicine breeds each (Sölkner et al., 2014). This region has been implicated in the control of parasitemia in cattle infected by *Trypanosoma congolense* (Hanotte et al., 2003), and is orthologous to the human chromosome segment 5q31-q33, known as the Th2 cytokine gene cluster, which has been shown to be implicated in the control of allergy and resilience against infectious diseases such as malaria (Garcia et al., 1998; Rihet et al., 1998; Flori et al., 2003; Hernandez-Valladares et al., 2004) and leishmaniasis (Jeronimo et al., 2007). The region also flanks *SPOCK1*, a candidate gene for puberty both in humans (Liu et al., 2009) and cattle (Fortes et al., 2010). Although fertility and resistance to infectious diseases are candidate biological drivers of this ROH hotspot, the gene and the phenotype underlying this putative selection signature are unknown.

The common ROH at 12:28433881-29743057 identified in the present study also overlaps a common ROH hotspot (Sölkner et al., 2014) and a region of divergent selection between *Bos taurus* and *Bos indicus* cattle (Gautier et al., 2009; Porto-Neto et al., 2013), and the segment encompasses the human ortholog *BRCA2*, involved in Fanconi anemia in humans (Howlett et al., 2002). A signature of selection nearby the 4:46384250-47113352 region detected here has also been reported by Gautier and Naves (2011), but the genes involved and the selective pressure remain uncharacterized.

## Conclusions

We used high-density SNP genotypes to successfully characterize autozygosity in Nellore cows under artificial selection for reproductive, carcass and weight traits. We have shown that, although the massive use of relatively few sires and artificial insemination has generated long stretches of homozygous haplotypes in the genomes of over 70% of these animals, inbreeding levels were considerably low in this population. We also found few genomic regions with high homozygosity across individuals, suggesting that the ongoing selection for reproductive, weight and carcass traits in this population is not very intensive or too recent to have left selection signatures in the form of ROH. Furthermore, the current common breeding practices of avoiding inbreeding in the mating schemes are antagonistic to additive trait selection, making it hard to maintain ROH signatures in the herds. The three candidate regions under selection identified herein were likely to be contributions from remote ancestors, predating the foundation of the Nellore breeding programs. The selective pressure effects and the genes involved in these regions should be subject of future investigation.

### Conflict of interest statement

The authors declare that the research was conducted in the absence of any commercial or financial relationships that could be construed as a potential conflict of interest.
