# Supplementary material for: Assessment of autozygosity in Nellore cows (Bos indicus) through high-density SNP genotypes
Source: Front Genet. 2015 Jan 29;6:5. doi: 10.3389/fgene.2015.00005 (PMC4310349; doi:10.3389/fgene.2015.00005)
Supplement: Supplementary file 1 [file DataSheet1.PDF]

Supplementary Material

Assessment of autozygosity in Nellore cows (*Bos indicus*) through high-density SNP genotypes

Ludmilla B. Zavarez$_{1}$[1], Yuri T. Utsunomiya$_{2}$[1], Adriana S. Carmo$_{3}$[1], Haroldo H.R. Neves$_{4}$[2,3], Roberto Carvalheiro$_{5}$[3], Maja Ferenčaković$_{6}$[4], Ana M. Pérez O'Brien$_{7}$[5], Ino Curik$_{8}$[4], John B. Cole$_{9}$[6], Curtis P. Van Tassell$_{10}$[6], Marcos V.G.B. da Silva$_{11}$[7], Tad S. Sonstegard$_{12}$[6], Johann Sölkner$_{13}$[5], José F. Garcia$_{14}$[1,8]*

[1]UNESP - Univ. Estadual Paulista, Faculdade de Ciências Agrárias e Veterinárias, Departamento de Medicina Veterinária Preventiva e Reprodução Animal, Jaboticabal, São Paulo, Brazil
[2]GenSys Consultores Associados, Porto Alegre, Rio Grande do Sul, Brazil
[3]UNESP - Univ. Estadual Paulista, Faculdade de Ciências Agrárias e Veterinárias, Departamento de Zootecnia, Jaboticabal, São Paulo, Brazil
[4]Department of Animal Science, Faculty of Agriculture, University of Zagreb, Zagreb, Croatia
[5]BOKU - University of Natural Resources and Life Sciences, Department of Sustainable Agricultural Systems, Division of Livestock Sciences, Vienna, Austria
[6]Animal Genomics and Improvement Laboratory, United States Department of Agriculture, Agricultural Research Service, Beltsville, Maryland, United States of America
[7]Bioinformatics and Animal Genomics Laboratory, Embrapa Dairy Cattle, Juiz de Fora, Minas Gerais, Brazil
[8]Laboratório de Bioquímica e Biologia Molecular Animal, UNESP – Univ. Estadual Paulista, Faculdade de Medicina Veterinária de Araçatuba, Departamento de Apoio, Produção e Saúde Animal, Araçatuba, São Paulo, Brazil

*** Correspondence:** José Fernando Garcia, Laboratório de Bioquímica e Biologia Molecular Animal, UNESP – Univ. Estadual Paulista, Faculdade de Ciências Agrárias e Veterinárias, Departamento de Apoio, Produção e Saúde Animal, Rua Clóvis Pestana 793, Araçatuba, São Paulo, 16050-680, Brazil.
jfgarcia@fmva.unesp.br

1. Methods for effective population size estimation

Effective population size (N_e) was estimated based on its approximate relationship with linkage disequilibrium (LD). More precisely, the expected squared correlation of allele frequencies at a pair of loci (r^2) can be related to N_e as follows:

$$E(r^2) = \frac{1}{(\alpha + kN_e c)} + var(r)$$

Where c is the linkage distance (in Morgans) between the loci, α and k are constants, and $var(r)$ is the chance disequilibrium variance introduced by experimental sampling. The analysis of autosomal markers (k = 4) under the assumption of absence of mutation (α = 1) and sampling error ($var(r)$ = 0) leads to the formula discovered by Sved (1971). Likewise, taking sampling error ($var(r)$ = 1/n, where n is the number of haplotypes), mutation (α = 2) and sex chromosomes into account (k = 4 for autosomes and k = 2 for sex chromosomes) leads to other known extensions of the problem (Hill, 1975; Weir & Hill, 1980). Based on this formula, Tenesa et al. (2007) introduced a nonlinear regression model to estimate N_e from genome-wide SNP data:

$$y_i = \frac{1}{(\alpha + \beta c_i)} + e_i$$

Where y_i = r^2 - 1/n is the LD for marker pair i corrected for the number of haplotypes, β is a parameter representing kN_e, c_i is the genetic distance between markers (in Morgans), and e_i is an error term. First, due to computational complexity, we used *PLINK v1.07* (Purcell et al., 2007) to calculate pairwise r^2 only for SNPs presenting MAF > 0.05 that were no more than 3 Mb apart. This distance constraint considered the rapid LD decay reported for the Nellore genome (McKay et al., 2007; Espigolan et al., 2013; Pérez-O'Brien et al., 2014). Following Uimari & Tapio (2010), only LD values of 0.01 < r^2< 0.99 were included, as the point estimates of N_e are infinite at r^2 = 0 and r^2 = 1. Second, the approximation c_i = $10^{-8}d_i$ was used, where d_i is the inter-marker distance in base pairs for SNP pair i, under the assumption of 1Mb ≈ 1cM. Third, the model above was fitted for each chromosome and α and β were estimated using nonlinear least squares in *R v3.1.1* (http://www.r-project.org/). Chromosomal N_e was derived as $\hat{N}_e = \hat{\beta}/4$ for autosomes and $\hat{N}_e = \hat{\beta}/2$ for the X chromosome. Finally, as the total number of computed r^2 values was too large to be regressed using available hardware (approximately 120 million), the genome-wide $\hat{N}_e$ was derived by fitting the nonlinear model using 1 million randomly sampled r^2 values from each autosome (summing up 29 million observations). Five replicates were processed in order to measure sampling variance.

2. Supplementary tables

Table S1. Parameter estimates for the nonlinear regression models by chromosome

Chromosome	Number of pairwise comparisons	$\hat{\alpha}$	SE($\hat{\alpha}$)	$\hat{\beta}$	SE($\hat{\beta}$)	$\widehat{Ne}$
1	7,154,155	4.052	0.003	1318.138	1.493	329.534
2	5,723,254	3.715	0.003	1479.120	1.779	369.780
3	6,155,446	4.321	0.004	1078.419	1.456	269.604
4	4,783,055	4.021	0.004	1597.914	2.206	399.478
5	5,128,268	4.085	0.004	1092.324	1.488	273.081
6	6,575,478	3.935	0.003	1233.294	1.362	308.323
7	5,172,412	3.594	0.003	1500.191	1.873	375.047
8	6,322,519	3.965	0.003	1251.309	1.440	312.827
9	5,380,061	3.710	0.003	1625.961	1.959	406.490
10	4,091,772	3.790	0.004	1840.789	2.673	460.197
11	4,564,182	3.768	0.003	1590.636	2.151	397.659
12	3,738,278	4.183	0.004	1382.465	2.224	345.616
13	3,506,642	3.852	0.004	1648.760	2.566	412.190
14	4,679,140	4.079	0.004	1326.500	1.753	331.625
15	3,428,608	4.048	0.004	1612.331	2.515	403.082
16	3,788,210	3.872	0.004	1553.210	2.245	388.302
17	4,031,419	4.901	0.005	798.770	1.468	199.692
18	2,969,957	3.578	0.004	1957.124	3.248	489.281
19	2,251,363	4.303	0.006	1599.284	3.219	399.821
20	3,133,031	3.969	0.004	1499.503	2.449	374.875
21	3,259,818	3.820	0.004	1395.949	2.355	348.987
22	2,583,455	4.138	0.005	1656.291	2.995	414.072
23	2,399,662	4.586	0.006	1718.008	3.305	429.502
24	2,822,059	3.850	0.005	1558.057	2.878	389.514
25	2,038,428	5.382	0.010	811.048	2.404	202.762
26	2,356,368	3.914	0.005	2029.060	3.904	507.265
27	1,832,751	4.534	0.007	1797.444	3.986	449.361
28	1,915,010	4.932	0.007	1279.928	3.002	319.982
29	1,830,764	4.319	0.007	1658.245	3.729	414.561
All autosomes	5 replicates of 29,000,000	4.099	0.004	1448.766	1.940	361.786
X	7,341,954	3.884	0.003	554.181	0.781	277.090

Table S2. Parameter estimates for the nonlinear regression model for 5 replicates of 1 million randomly sampled r^2 values from each autosomal chromosome.

Autosome replicate	$\hat{\alpha}$	$\hat{\beta}$	$\widehat{N}e$
1	4.097	1447.460	361.865
2	4.101	1446.598	361.649
3	4.108	1444.103	361.026
4	4.096	1448.812	362.203
5	4.097	1448.766	362.192
average	4.100	1447.148	361.787
stdev	0.005	1.941	0.485

3. Supplementary figures

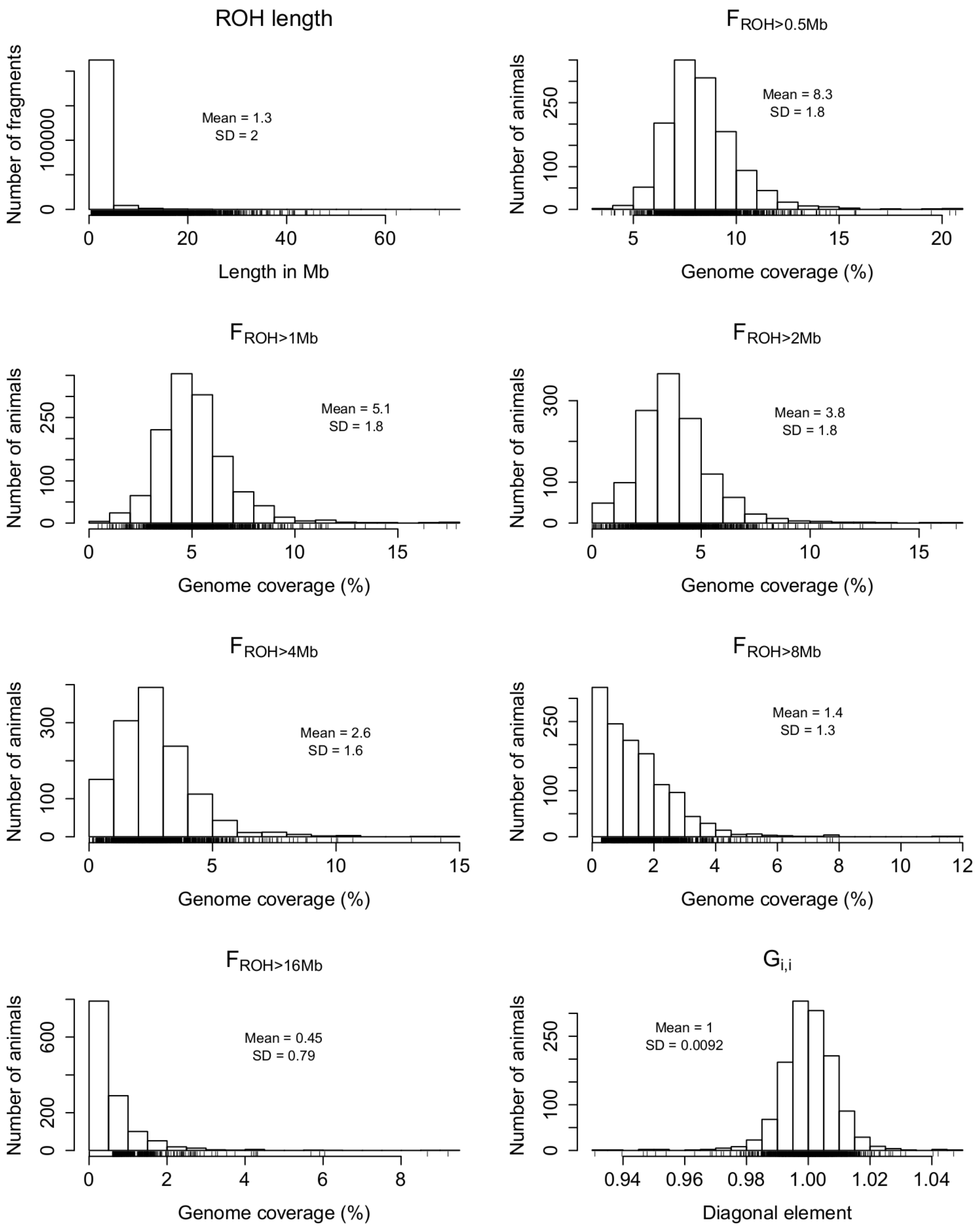

Figure S1. Frequency distributions of all detected runs of homozygosity (ROH) across samples, percentage of the total genome (autosomes + X) coverage by ROH (F_{ROH}) of different minimum lengths (>0.5, >1, >2, >4, >8 and >16 Mb), and diagonal elements of the realized genomic relationship matrix ($G_{i,i}$).

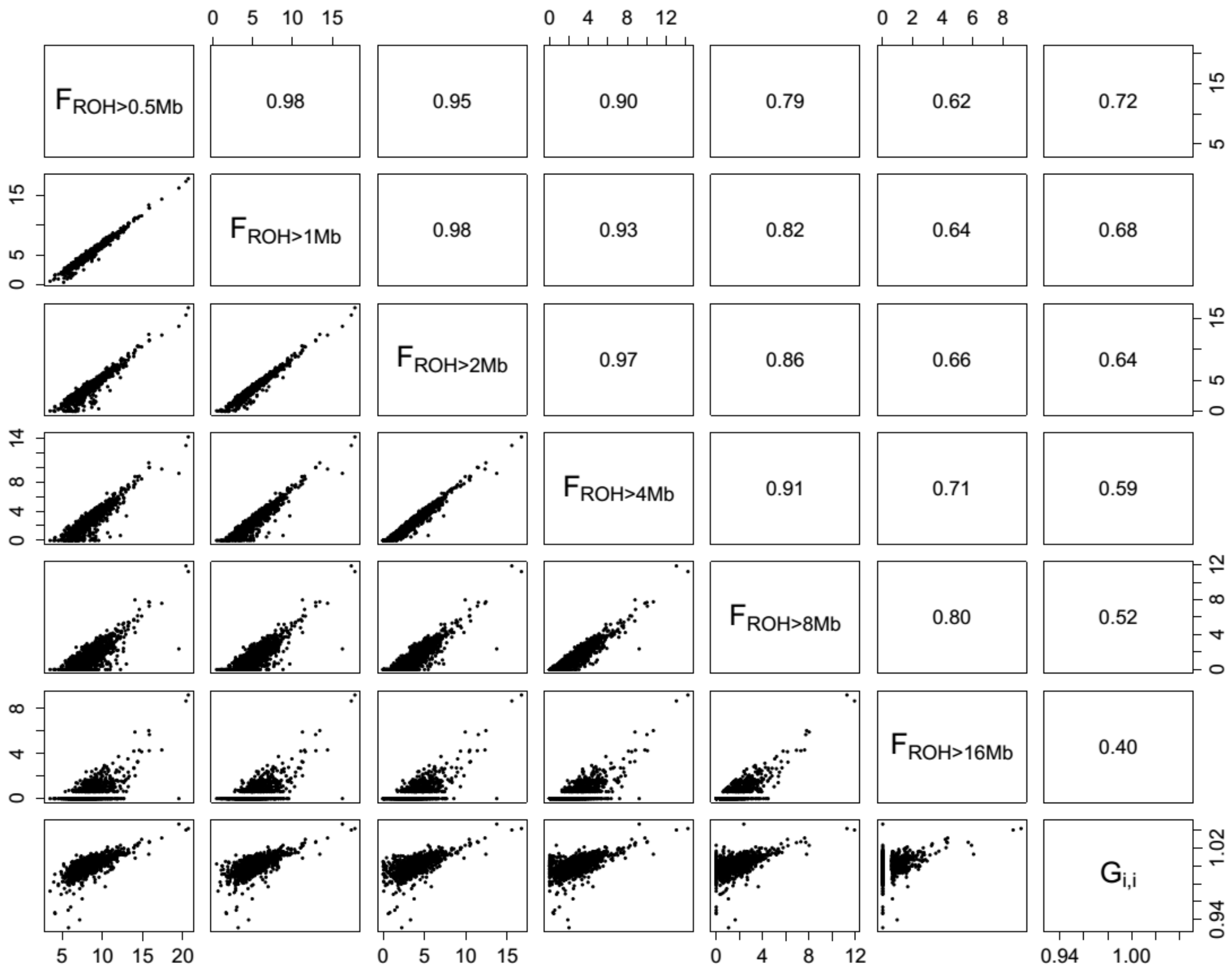

Figure S2. Scatterplots (lower panel) and correlations (upper panel) of percentage of total genome (autosomes + X) coverage by runs of homozygosity (F_{ROH}) of different minimum lengths (>0.5, >1, >2, >4, >8 and >16 Mb) and diagonal elements of the realized genomic relationship matrix ($G_{i,i}$). The last column of panels on the right indicates that the correlation between F_{ROH} and $G_{i,i}$ decreases as a function of minimum fragment size.

4. References

Espigolan, R.; Baldi F.; Boligon, A. A.; Souza, F. R.; Gordo, D. G.; Tonussi, R. L.; Cardoso, D. F.; Oliveira, H. N.; Tonhati, H.; Sargolzaei, M.; Schenkel, F. S.; Carvalheiro, R.; Ferro, J. A.; Albuquerque, L. G. (2013). Study of whole genome linkage disequilibrium in Nellore cattle. *BMC Genomics*. 14, 305.

Hill, W.G. (1975). Linkage disequilibrium among multiple neutral alleles produced by mutation in finite population. *Theor. Popul. Biol.* 8, 117-126.

McKay, S. D.; Schnabel, R. D.; Murdoch, B. M.; Matukumalli, L. K.; Aerts, J.; Coppieters, W.; Crews, D.; Neto, E. D.; Gill, C. A.; Gao, C.; Mannen, H.; Stothard, P.; Wang, Z.; Van Tassell, C. P.; Williams, J. L.; Taylor, J. F.; Moore, S. S. (2007). Whole genome linkage disequilibrium maps in cattle. *BMC Genet*. 8, 74.

Pérez O'Brien, A. M.; Mészáros, G.; Utsunomiya, Y. T.; Sonstegard, T. S.; Garcia, F. J.; Van Tassell, C. P.; Carvalheiro, R.; Da Silva, M. V. B.; Sölkner, J. (2014). Linkage disequilibrium levels in Bosindicus and Bostaurus cattle using medium and high density SNP chip data and different minor allele frequency distributions. *Livest. Sci*. 166, 121-132.

Purcell, S.; Neale, B.; Todd-Brown, K.; Thomas, L.; Ferreira, M. A.; Bender, D.; Maller, J.; Sklar, P.; de Bakker, P. I.; Daly, M. J.; Sham, P. C. (2007). PLINK: a tool set for whole-genome association and population-based linkage analyses. *Am. J. Hum. Genet*. 81(3), 559-575.

Tenesa, A.; Navarro, P.; Hayes, B. J.; Duffy, D. L.; Clarke, G. M.; Goddard, M. E.; Visscher, P. M. (2007). Recent human effective population size estimated from linkage disequilibrium. *Genome Res.* 17(4), 520-526.

Uimari, P. and Tapio, M. (2011). Extent of linkage disequilibrium and effective population size in Finnish Landrace and Finnish Yorkshire pig breeds. *J. Anim. Sci*. 89, 609-614.

Sved, J. A, (1971). Linkage disequilibrium and homozygosity of chromosome segments in finite populations. *Theor. Popul. Biol*. 2, 125-141.

Weir, B. S.; Hill, W.G. (1980). Effect of mating structure on variation in linkage disequilibrium. *Genetics*. 95, 477-488.
